# Association between the beta‐blockers, calcium channel blockers, all‐cause mortality and length of hospitalization in patients with heart failure with preserved ejection fraction: A meta‐analysis of randomized controlled trials

**DOI:** 10.1002/clc.24058

**Published:** 2023-06-04

**Authors:** Mingming Wu, Dan Ni, Lin‐ling Huang, Shengjun Qiu

**Affiliations:** ^1^ Department of Cardiology Jiangsu Rudong County People's Hospital Nantong Jiangsu China; ^2^ Department of Geriatrics Meishan People's Hospital Sichuan Meishan China; ^3^ Department of Medical College Wuhan Railway Vocational College of Technology Wuhan Hubei China

**Keywords:** beta‐blockers, calcium channel blockers, cardiovascular outcomes, heart failure with preserved ejection fraction, left ventricular ejection fraction

## Abstract

**Purpose:**

To establish an association between beta‐blockers (BBs), calcium channel blockers (CCBs), all‐cause mortality, and hospitalization in patients with Heart failure with preserved Ejection Fraction (HFpEF).

**Methods:**

The present meta‐analysis has been performed as per the guidelines of (PRISMA). An inclusive literature search was made without any limitations on language using the electronic databases Cochrane Library, EMBASE, and PubMed up to November 2022. The outcomes evaluated in this meta‐analysis involved all‐cause mortality and hospitalization due to heart failure. The number of patients with HFpEF and their positive outcomes was extracted and analyzed using RevMan software.

**Results:**

In total, 10 articles were included in the present meta‐analysis, with a pooled sample size of 12 940 HFpEF patients. In comparison with placebo, both BB and CCB substantially reduced the risk of all‐cause mortality and hospitalization. However, BB are more effective because they provide a significant reduction in all‐cause mortality (risk ratio (RR) = 0.60; 95% confidence interval [CI] = 0.43–0.83; *p* = .002] and hospitalization (RR = 0.54; 95% CI = 0.37–0.80; *p* = .002) as compared with CCB with a risk ratio of all‐cause mortality (RR = 0.77; 95% CI = 0.60–0.98; *p* = .03) and hospitalization (RR = 0.63; 95% CI = 0.44–0.90; *p* < .00001). A random‐effects model was used because of high heterogeneity between the studies (*I* ^2^ > 70%).

**Conclusions:**

The current meta‐analysis suggests that BBs were more beneficial than CCB in reducing all‐cause mortality and hospitalization duration in patients with HFpEF.

## Graphical abstract

Beta‐blockers versus calcium channel blockers.

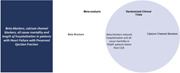

## 1 INTRODUCTION

Heart failure with preserved left ventricular (LV) ejection fraction (HFpEF) is defined as the presence of heart failure in the absence of any evidence of reduced LV ejection fraction.^1^, ^2^ This type of heart failure is also known as heart failure with intact left ventricular (LV) function. The incidence of HFpEF is on the rise, and research has linked it to an increase in the number of patients who require hospitalization.^3^ Numerous studies on patients with chronic heart failure have shown that this syndrome has a high mortality and morbidity rate.^4^ However, evidence from clinical studies revealing improvements in mortality has been variable and essentially unbiased; a few investigations suggest that pharmacological therapy may improve the tolerance for exercise and quality of life of these people.^5^ Given that patients with heart failure with intact left ventricular ejection fraction (LVEF) are more likely to be older and have a greater number of comorbidities than their counterparts,^6^, ^7^ the efficacy of pharmacological treatment may be best assessed by its impact on hospitalization and associated symptoms. This condition can be treated with neprilysin inhibitors, sacubitril, valsaltran, an interleukin‐1 blocker, Spironolactone, a RAAS blocker, a beta‐blocker (BB), Sildenafil, an aldosterone antagonist, empagliflozin, and calcium channel blockers (CCBs).^7^, ^8^, ^9^, ^10^ Even though there is a paucity of evidence supporting their advantages, BBs are often recommended for HFpEF. BBs, or beta‐adrenergic blocking medications, works by inhibiting the release of stress chemicals adrenaline and noradrenaline in specific regions, which decreases the heart rate and the force with which blood is pushed throughout the body. In their research articles, Yum et al.^11^ and Xu et al.^12^ found that over 80% of patients with HFpEF received BBs. However, current research suggests a link between the use of CCBs and better outcomes in individuals with HFpEF. CCBs improve HFPEF by reducing peripheral vasoconstriction and, thus, lowering left ventricular afterload. For instance, Vider et al.^13^ reported in their study article that the use of CCBs was related to reduced risks of death in patients with HFpEF, whereas Borlaug et al.^14^ observed that CCBs were the third‐ or fourth‐line therapy for hypertension in patients with HFpEF. Due to conflicting or insufficient information concerning the effects of medications in patients with HFpEF, it is essential to examine the efficacy of both current treatment choices to avert unembellished outcomes in patients with HFpEF. There is an immediate need for treatment of heart failure with preserved ejection fraction (HFpEF), yet there are currently no medications that have been shown to be successful. Patients with HFpEF routinely get BBs despite a lack of clinical evidence supporting their use. Recent clinical trials have shown that more than 75% of patients with HFpEF are being treated with BBs. Similarly, recent studies suggested that HFpEF patients who used CCBs had better results. In this meta‐analysis, we sought to examine the impact of BBs and CCBs on all‐cause mortality and hospitalization due to cardiovascular causes among patients with heart failure and preserved LVEF.

## 2 MATERIALS AND METHODS

The present meta‐analysis was undertaken following the Preferred Reporting Items of Systematic Reviews and Meta‐Analyses (PRISMA) guidelines.

### 2.1 Data sources and searches

An inclusive literature search was conducted without any limitations on the year and language of publication utilizing the electronic databases Cochrane Library, EMBASE, and PubMed up to September 30, 2022 using the following search criteria: (I) “heart failure with preserved ejection fraction “ OR “HFpEF”; (II) “beta‐blockers” OR BB; (III) “calcium channel blockers” OR “CCB”; (IV) “all‐cause mortality”; (V) Length of hospitalization; (VI) and “cardiovascular outcomes.” Within the context of the search strategy, the Boolean operator “AND” was used to combine the Medical Subject Headings (MeSH) with the text keywords. First, duplicate articles were deleted from the search results, followed by a title and abstract screening of the remaining articles. Finally, the full texts of all the qualified studies were retrieved and reviewed for inclusion and exclusion based on the inclusion–exclusion criteria.

### 2.2 Study selection

The literature search was conducted separately by two authors. Through discussion, a consensus was obtained in the event of dispute. The following conditions must be met for a study to qualify: (a) randomized controlled trials (RCTs) examining the efficacy of BBs and CCBs; (b) studies that evaluate at least one of the following two outcomes: all‐cause mortality, or hospitalization for cardiovascular causes. Exclusion criteria included clinical trials with a follow‐up time of smaller than one month. Studies that were led on healthy volunteers or on those who suffered from disorders other than HFpEF were also not considered. In the end, studies that compared medications other than BBs and CCBs were not included in this meta‐analysis.

### 2.3 Data extraction

A computerized data extraction form was developed in Microsoft Excel and utilized for the purpose of listing the fundamental information of the studies^15^, ^16^, ^17^, ^18^, ^19^, ^20^, ^21^, ^22^, ^23^, ^24^ selected for the meta‐analysis. This included the first author's name, the year of publication, the intervention, the sample size in each group, the duration of follow‐up, and the outcomes. The data were extracted by two different authors in an independent process, and then the results of both authors' extractions were compared. In the event that there was a difference of opinion, a consensus was reached through debate. Third author was also included in the event whenever necessary.

### 2.4 Quality assessment of the included studies

The Cochrane Risk of Bias tool was applied to evaluate the methodological validity of each and every study that was incorporated into the meta‐analysis. During the process of data extraction, selected articles were given a score, and the RevMan version 5.4 software^25^ was used to construct a quality evaluation graph.

### 2.5 Data analysis

For the process of data analysis RevMan version 5.4.0 and MedCalc software^26^ were utilized. For the purpose of assessing the pooled risk ratio in addition to the 95% confidence interval (CI) for both two outcomes, the Mantel–Haenszel technique with the random effect model^27^ was utilized. A statistically significant result was regarded to have a *p*‐value of < .05.^28^ To graphically depict the risk ratio along with the 95% CI, forest plots^29^ were utilized. *I* ^2^ statistics were utilized to ascertain the degree of heterogeneity present among the study's findings,^30^ and for comparison of BBs and CCBs in terms of all‐cause mortality, and length of hospitalization was done using meta‐regression analysis.

## 3 RESULTS

### 3.1 Literature search results

Figure S1 depicts the PRISMA chart for the selection of research. Through a comprehensive search of online databases, 567 studies were identified in total. After eliminating duplicates, the abstracts and titles of 376 studies were screened. Only 48 studies qualified for full‐text evaluation. Ten publications were finally included in the present meta‐analysis based on the PICOS criteria shown in Table S1.^31^

The main characteristics of all included trials, including 12 940 patients with HFpEF are displayed in Table 1. Five publications evaluated the effectiveness of BBs,^15^, ^16^, ^19^, ^20^, ^24^ while five articles evaluated the effectiveness of CCBs.^17^, ^18^, ^21^, ^22^, ^23^ In all included investigations, the median follow‐up time ranged from 1 month to 42 months.

**Table 1:** Characteristics of included studies.

Study	Year	Intervention	Drug	Sample size	Age of patients	Follow‐up	Primary outcomes
Bergstrom et al.^15^	2004	Beta‐blocker	carvedilol	97	40–80 years	6 months	All‐cause mortality, Length of Hospital stay
Gomez et al.^16^	2009	Beta‐blocker	carvedilol	1085	55–75 years	12 months	All‐cause mortality, Length of Hospital stay
Kiuchi et al.^17^	2017	Calcium channel blocker	Azelnidipine	25	>18 years	6 months	All‐cause mortality, Length of Hospital stay
Oikawa et al.^18^	2010	Calcium channel blocker	Nifedipine	30	40‐80 years	1 month	All‐cause mortality, Length of Hospital stay
Palau et al.^19^	2013	Beta‐blocker	Amlodipine	1654	>18 years	33 months	All‐cause mortality, Length of Hospital stay
Patel et al.^20^	2019	Beta‐blocker	carvedilol	3759	>65 years	6 months	All‐cause mortality, Length of Hospital stay
Patel K et al.^21^	2014	Calcium channel blocker	Amlodipine	1620	>65 years	6 years	All‐cause mortality, Length of Hospital stay
Tsutsui et al.^22^	2010	Calcium channel blocker	Amlodipine	985	50–85 years	12 months	All‐cause mortality, Length of Hospital stay
Wang et al.^23^	2021	Calcium channel blocker	Amlodipine	3440	60–80 years	3.5 years	All‐cause mortality, Length of Hospital stay
Yamamoto et al.^24^	2013	Beta‐blocker	carvedilol	245	60–80 years	3.2 years	All‐cause mortality, Length of Hospital stay

### 3.2 Assessment of risk of bias and publication bias

**Figure 1:**
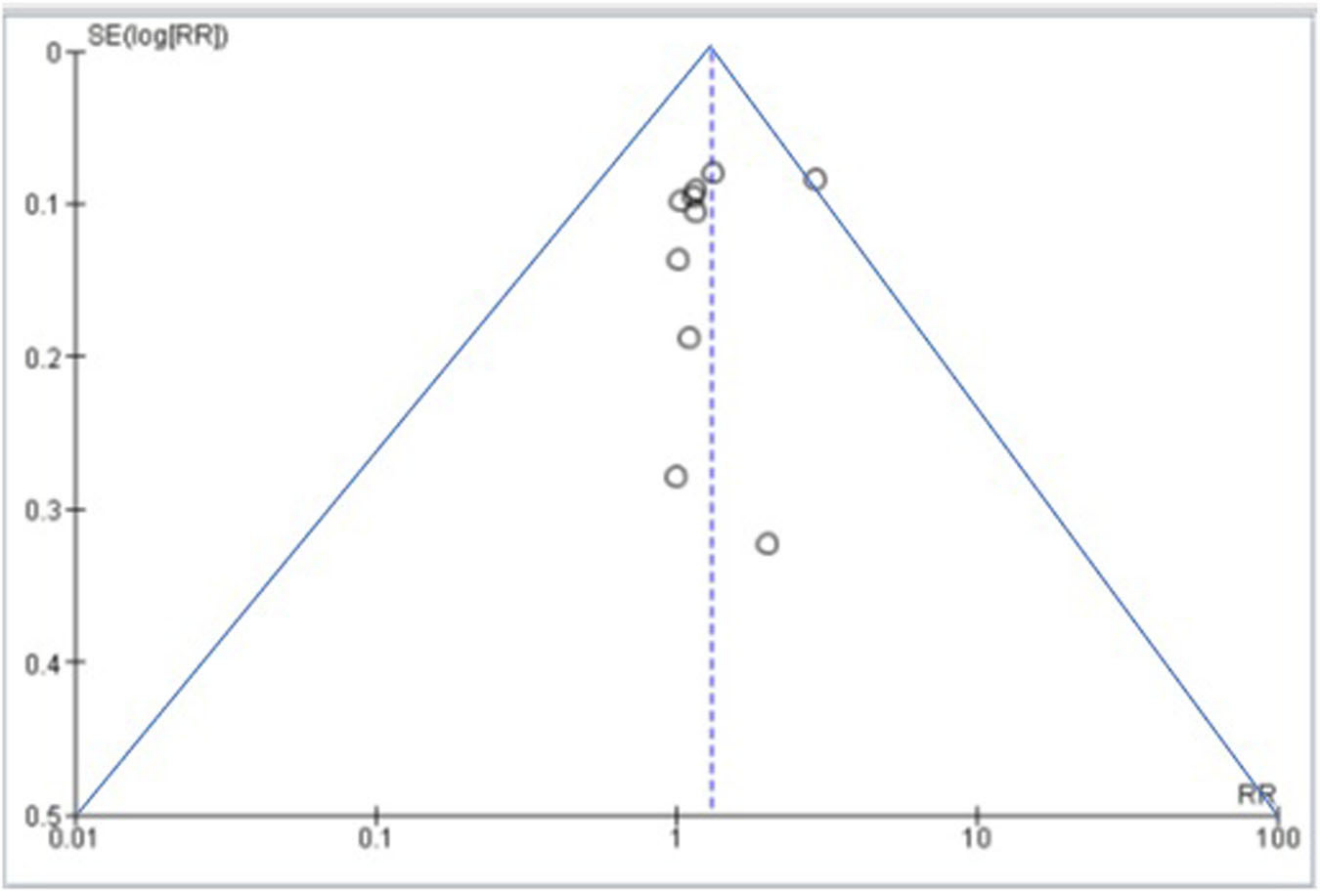
Funnel plot.

A pre‐designed questionnaire was used for assessment of risk of bias and results are shown in Table S2. Figure S2 depicts the risk of bias summary, whereas Figure S3 depicts the risk of bias graph. Five of the 10 included studies were associated with low risk of bias whereas; three had a moderate risk attributable to allocation concealment and selective reporting. The other two studies posed a high risk of reporting bias. Figure 1 depicts the funnel plot, which indicated a low probability of publication bias with a significant *p*‐value of .583 for Begg's test.^32^

### 3.3 Efficacy outcomes

**Figure 2:**
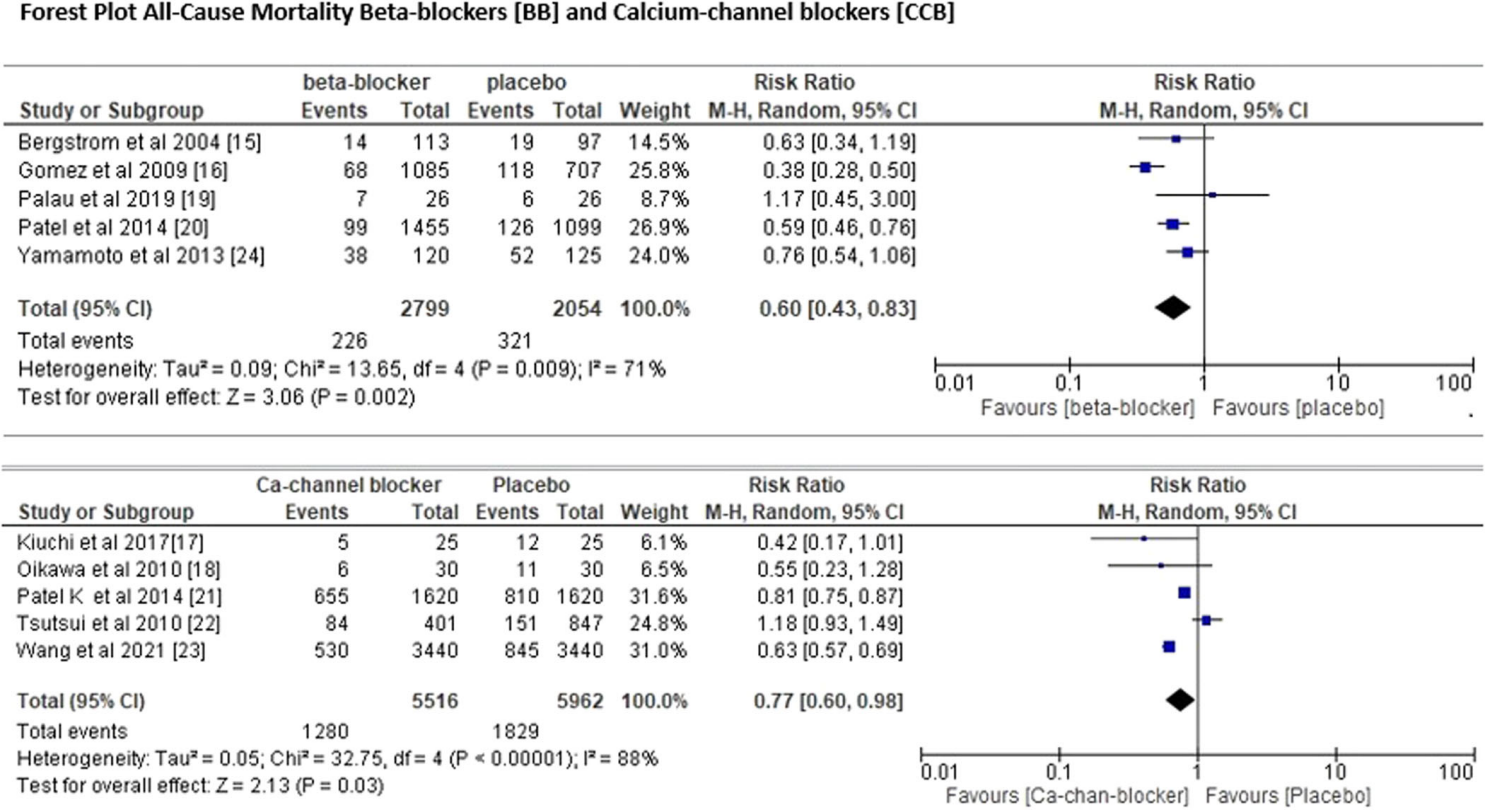
Forest plot for all‐cause mortality. CI, confidence interval.

**Figure 3:**
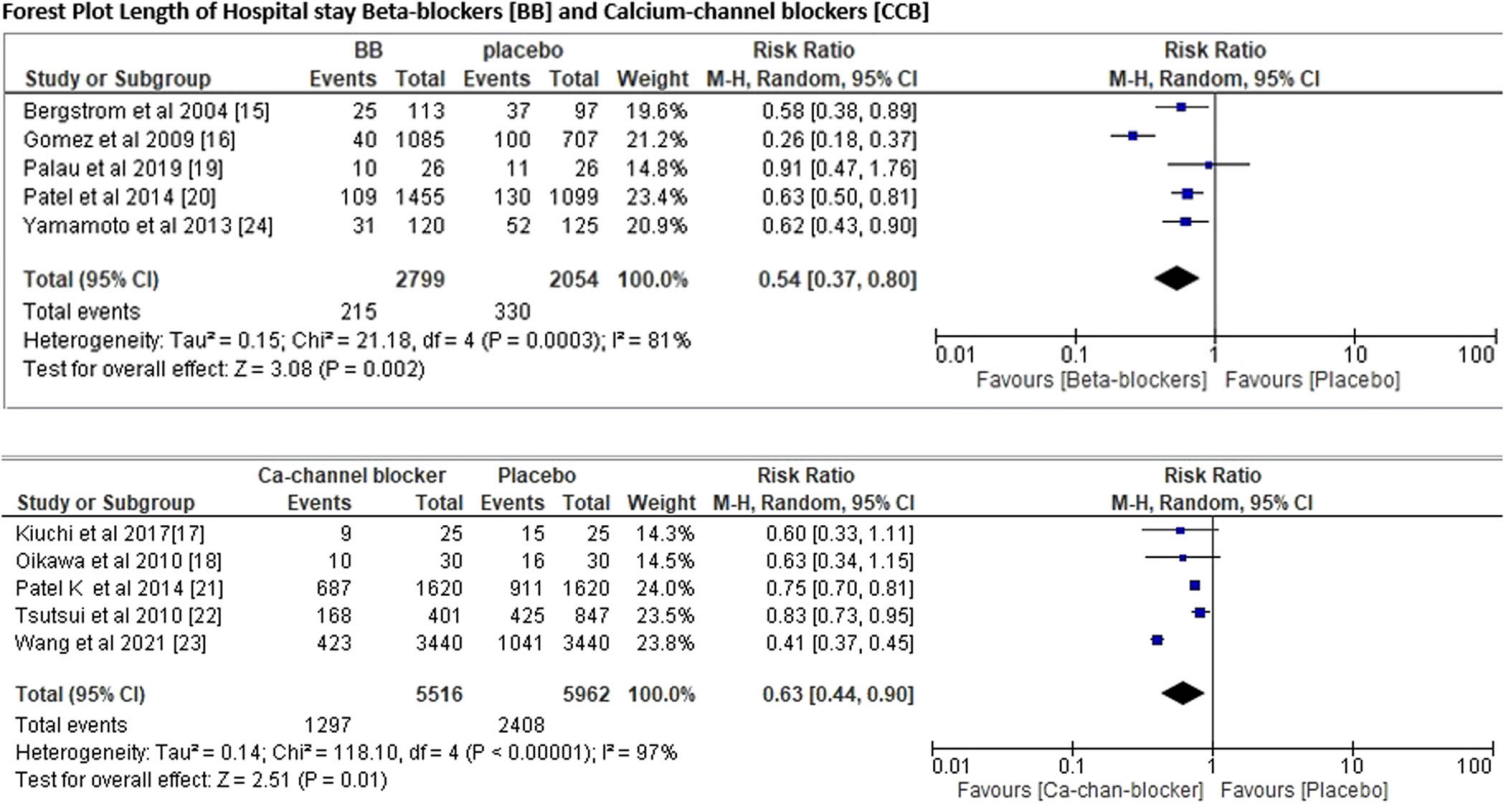
Forest plot for length of hospital stay. CI, confidence interval.

**Figure 4:**
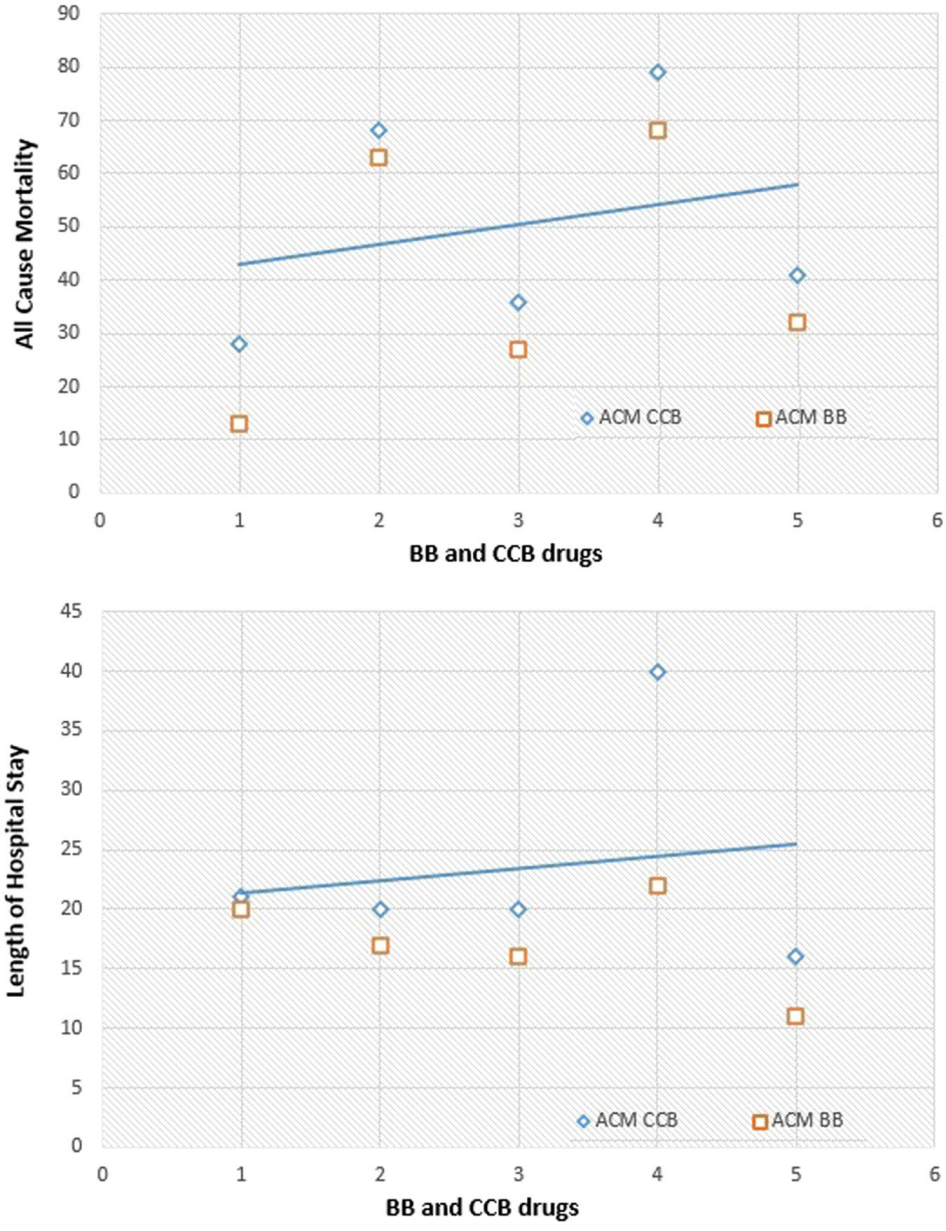
Regression analysis of beta‐blocker (BB) versus calcium channel blocker (CCB).

All 10 trials with a total of 12 940 individuals provided data on all‐cause mortality and length of hospitalization. Figure 2 depicts the comprehensive pairwise assessment of each of the treatment groups (BBs and CCBs] with placebo for all‐cause mortality. Both BBs and CCBs significantly lowered the chance of mortality from any cause as compared with placebo. The pooled RR for BBs was (RR = 0.60; 95% CI = 0.43–0.83; *p* = .002) and *I* ^2^ value of 71%, whereas the pooled RR for CCBs was (RR = 0.77; 95% CI = 0.60–0.98; *p* = .03) and *I* ^2^ value of 88%. For length of hospitalization risk ratio of BBs was (RR = 0.54; 95% CI = 0.37–0.80; *p* = .002), *I* ^2^ value of 81% as compared with CCB with risk ratio of hospitalization (RR = 0.63; 95% CI = 0.44–0.90; *p* < .00001), *I* ^2^ value of 97% (Figure 3). The comparison of BBs with CCBs for all‐cause mortality and length of hospitalization was done via meta‐regression analysis as shown in Figure 4, and we found that BBs were more effective than CCBs, since the risk of all‐cause death was 45% lesser in patients who took BBs as compared with those patients who took CCBs. Nevertheless, BBs are more effective than CCBs, as hospitalization for cardiac reasons was 73% lower in patients getting BBs as opposed to CCBs.

## 4 DISCUSSION

Since heart failure is a leading cause of death worldwide, it's a matter of grave medical concern to understand all of its facets. With a greater understanding of the best medication, its mortality and morbidity can be minimized, and the overall survival rate can be improved. According to the guidelines published by the European Society of Cardiology, it is essential to lessen the problem of readmission for those patients who have HFpEF.^33^ Due to the fact that patients with HFpEF (heart failure with preserved ejection fraction) have a higher probability of being older than those with HFrEF, they are more likely to be hospitalized for cardiac reasons, which is linked with an inferior quality of life and an advanced mortality rate.^34^, ^35^ The selection of effective medicinal treatments for HFpEF patients remains a significant challenge for medical professionals.

HFpEF is mostly associated with the aberrant diastolic activity of the left ventricle (LV) function and an increase in arterial stiffness, both of which will lead to an improper ventricular–arterial (V–A) coupling. Both of these factors may be prevented. HFpEF patients had decreased aortic distensibility and flow‐mediated vasodilation. Endothelial dysfunction due to decreased NO bioavailability causes vascular dysfunction in both distal and proximal arteries. This stiffness, as well as a reduction in arterial compliance, cause an abnormal vasodilator reaction to exercise, which increases pulse pressure waves and blood flow abnormalities that impair left ventricle diastolic function and reduce peripheral‐mediated vasodilation and blood flow to skeletal muscle.^36^, ^37^ The pathophysiology of HFpEF is complex, with multiple causes coexisting in a single individual to cause symptomatic heart failure.^38^ The possible pathophysiological processes include the thickness of the left ventricular wall, extension of left atrium, higher ventricular filling pressure, right ventricular failure, pulmonary vascular disease, and expansion in the volume of plasma.^39^, ^40^, ^41^ The primary contributor to the pathophysiology of HFpEF is tissue congestion, which is caused by higher heart‐filling pressures. BBs have been shown to improve left ventricular (LV) hypertrophy and fibrosis, as well as reduce inflammatory alterations and oxidative stress in HFpEF patients, increasing their survival rates.^42^, ^43^ The most recent recommendations for the treatment of HFpEF include the following: controlling the volume status with a proper dose of diuretics; controlling hypertension; treating contributory risk factors such as insomnia, cardiovascular disease, and valve disease; and nutritional education.

BBs were related to a significant reduction in all‐cause mortality in this investigation, which was reported on the basis of our complete meta‐analysis of the available RCTs conducted on patients with HFpEF (heart failure with preserved ejection fraction). Although CCBs have a significant influence on all‐cause mortality and hospitalization, BBs have a larger positive effect on HFpEF patient outcomes. Fukata et al.^44^ performed a meta‐analysis that included both observational studies and RCTs and reported in their meta‐analysis that use of BBs reduces the risk of all‐cause mortality (RR = 0.80 (95% CI = 0.61–1.05, *p* 0.001). Similarly, Xu et al.^45^ found that blocker treatment was associated with a significant reduction in all‐cause mortality (RR = 0.73; 95% CI = 0.65–0.82, *p* 0.001] in their meta‐analysis. Similarly, Kovacevic et al.^46^ and Shields et al.^47^ that CCBs are effectives drugs for HFpEF. Similar to these findings, we found that the use of BB and CCB for HFpEF significantly reduced the risk of all‐cause mortality and hospitalization compared with placebo. Patients with HFpEF would benefit more from treatment with BBs than CCBs, according to this meta‐analysis.

## 5 LIMITATIONS

The present meta‐analysis has a number of limitations, such as a lack of data comparing different therapies; hence, we were unable to compare the therapies. Another restriction is the definition of HFpEF, which in some research is defined as heart failure with LVEF below 50% and in many trials as heart failure with LVEF between 40% and 49%. Aside from this, risk ratio values were predominantly used to identify the relationship between medicines, which may induce bias when comparing the results of RCTs of varied lengths. More RCTs with strict inclusion criteria for ejection fraction and comparisons of different medicines in terms of functional capacity, quality of life, and cardiovascular outcomes in HFpEF patients are needed in the future.

## 6 CONCLUSIONS

When compared with CCBs, the results of this meta‐analysis of randomized controlled trials involving HFpEF patients showed that the BB drugs considerably reduced the risk of mortality from any cause as well as the need for hospitalization among these patients. This meta‐analysis concluded that BBs were more effective in HFpEF patients based on statistically significant results.

## AUTHOR CONTRIBUTIONS

Mingming Wu: Concept and designed the study. Dan Ni: analyzed data and drafting of the manuscript. Lin‐ling Huang: Collected the data and helped in data analysis. Shengjun Qiu: Proofreading and final editing along with guarantor of the manuscript. All authors read and approved the final version of the manuscript.

## CONFLICT OF INTEREST STATEMENT

The authors declare that they have no conflict of interest.

## ETHICS STATEMENT

Ethical approval was not required as this study was based on publicly available data.

## Supporting information

Supplementary Figure 1: Study Flow Diagram.

Supplementary Figure 2: Risk of Bias Summary.

Supplementary Figure 3: Risk of Bias Graph.

Supporting information.

Supporting information.

## Data Availability

All data generated or analyzed during this study are included in this article. Further inquiries can be directed to the corresponding author.
