# Supplementary figures and images for: Association between the beta‐blockers, calcium channel blockers, all‐cause mortality and length of hospitalization in patients with heart failure with preserved ejection fraction: A meta‐analysis of randomized controlled trials
Source: Clin Cardiol. 2023 Jun 4;46(8):845–52. doi: 10.1002/clc.24058 (PMC10436801; doi:10.1002/clc.24058)

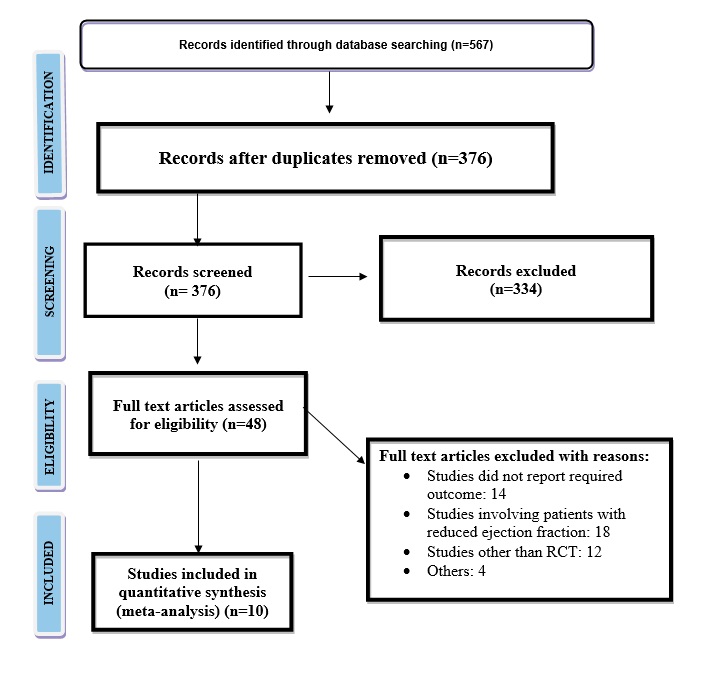

Supplement: Supplementary file 1 — Supplementary Figure 1: Study Flow Diagram. [file CLC-46-845-s002.jpg]

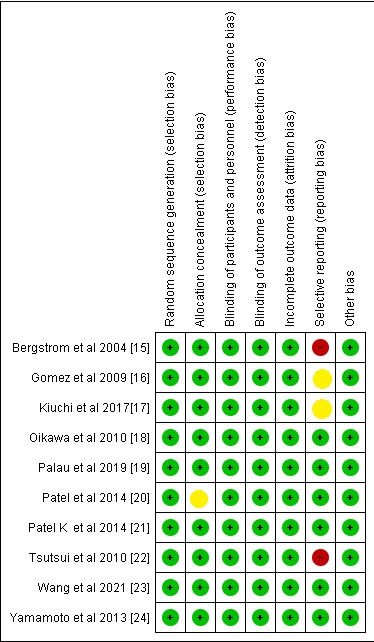

Supplement: Supplementary file 2 — Supplementary Figure 2: Risk of Bias Summary. [file CLC-46-845-s003.jpg]

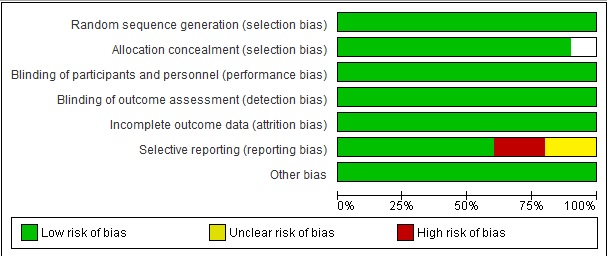

Supplement: Supplementary file 3 — Supplementary Figure 3: Risk of Bias Graph. [file CLC-46-845-s001.jpg]
