# Supplementary material for: Association between the beta‐blockers, calcium channel blockers, all‐cause mortality and length of hospitalization in patients with heart failure with preserved ejection fraction: A meta‐analysis of randomized controlled trials
Source: Clin Cardiol. 2023 Jun 4;46(8):845–52. doi: 10.1002/clc.24058 (PMC10436801; doi:10.1002/clc.24058)
Supplement: Supplementary file 4 — Supporting information. [file CLC-46-845-s004.docx]

**S. Table 1: PICOS Search**

| **P (Patient, Problem, Population)** | Patients of Heart failure with preserved ejection fraction [HFpEF] |
| --- | --- |
| **I (Intervention)** | The Efficacy of Beta -blockers versus Calcium channel Blockers on long-term outcomes in patients with HFpEF |
| **C (Comparison, control or comparator)** | Beta -blockers versus Calcium channel Blockers |
| **O [outcome (s)]** | Beta -blockers are more effective and safer for the treatment of patients with HFpEF |
| **S (study type)** | Randomized controlled trials |
