# Supplementary material for: Association between the beta‐blockers, calcium channel blockers, all‐cause mortality and length of hospitalization in patients with heart failure with preserved ejection fraction: A meta‐analysis of randomized controlled trials
Source: Clin Cardiol. 2023 Jun 4;46(8):845–52. doi: 10.1002/clc.24058 (PMC10436801; doi:10.1002/clc.24058)
Supplement: Supplementary file 5 — Supporting information. [file CLC-46-845-s005.docx]

**S. Table 2: Risk Assessment for Included Studies**

| **Study ID and Year** | **Bergstrom et al [15]** | **Gomez et al [16]** | **Kiuchi et al [17]** | **Oikawa et al [18]** | **Palau et al [19]** | **Patel et al [20]** | **Patel K et al [21]** | **Tsutsui et al [22]** | **Wang et al [23]** | **Yamamoto et al [24]** |
| --- | --- | --- | --- | --- | --- | --- | --- | --- | --- | --- |
| Was a consecutive or random sample of patients enrolled? | Y | Y | Y | Y | Y | Y | Y | Y | Y | Y |
| Did the study avoid inappropriate exclusions | Y | Y | Y | Y | Y | Y | Y | Y | Y | Y |
| Did all patients receive the same reference standard | Y | Y | Y | Y | Y | Y | Y | Y | Y | Y |
| Were all patients included in the analysis | N | N | N | N | N | N | N | N | N | N |
| Was the sample frame appropriate to address the target population? | Y | Y | Y | Y | Y | Y | Y | Y | Y | Y |
| Were study participants sampled in an appropriate way? | Y | Y | Y | Y | Y | Y | Y | Y | Y | Y |
| Were the study subjects and the setting described in detail? | Y | Y | Y | Y | Y | Y | Y | Y | Y | Y |
| Were valid methods used for the identification of the condition? | Y | Y | Y | Y | Y | Y | Y | Y | Y | Y |
| Was the condition measured in a standard, reliable way for all participants? | Y | Y | Y | Y | Y | Y | Y | Y | Y | Y |
